# Assessment of knowledge and attitudes toward human papillomavirus and its vaccination among female nursing students at Umm Al-Qura University, Saudi Arabia

**DOI:** 10.3389/fgwh.2025.1669950

**Published:** 2025-12-18

**Authors:** Mona Seleh Alwadai, Nawal Abdulghani

**Affiliations:** Faculty of Nursing, Umm Al-Qura University, Makkah, Saudi Arabia

**Keywords:** human papillomavirus, HPV, nursing students, Makkah, awareness, attitude, community

## Abstract

**Background:**

Human papillomavirus (HPV) is the most common sexually transmitted infection in women worldwide. Due to the high prevalence, communities need to know that the risk of HPV infection can be prevented by HPV vaccination.

**Aim:**

This study aims to assess the knowledge and attitudes of female nursing students at Umm Al-Qura University regarding HPV infection and HPV vaccination.

**Materials and methods:**

A descriptive cross-sectional quantitative study was conducted among 261 female nursing students using a self-administered online questionnaire. Data were collected through the validated 28-item Knowledge and Attitudes Toward HPV and Its Vaccination for University Students Scale, which was adapted and culturally modified to align with the Saudi context. The instrument comprised three parts: demographic information, general knowledge, and attitudes toward HPV vaccination. A non-probability purposive sampling technique was used. Data were analyzed using descriptive statistics and one-way ANOVA to explore differences across demographic variables.

**Results:**

Of the 261 participants, 49.4% demonstrated poor knowledge, 47.1% moderate knowledge, and 3.4% high knowledge. The mean attitude score was 2.79 ± 0.87, indicating a moderate level of acceptance toward HPV vaccination. Although most participants recognized its preventive importance, barriers remained, primarily related to parental consent and communication. One-way ANOVA results showed no significant differences in knowledge based on academic year or parental education, whereas living arrangement demonstrated a significant effect (*F* = 2.826, *p* = 0.025, *η*^2^ = 0.042).

**Conclusion:**

Nursing students had low to moderate knowledge of HPV and held a moderate level of acceptance toward the HPV vaccine highlighted the needs for targeted interventions and educational initiatives to enhance HPV vaccination uptake and promote overall well-being in the community.

## 1 Introduction

### 1.1 Research background

Human Papillomavirus (HPV) is a common sexually transmitted infection that can be the primary cause to different type of cancers, including cervical cancer (1). In Saudi Arabia, cervical cancer is the second most common cancer among women, and HPV is the most common cause of cervical cancer in Saudi women of reproductive age (2). Each year, more than 12,000 females in the United States are diagnosed with cervical cancer due to HPV, and more than 4,000 women succumb to the disease (3).

In recent years, HPV diagnoses and the associated health consequences have increased worldwide. Over 90% of genital, vaginal, cervical, vulvar, and oropharyngeal cancers caused by HPV can be prevented through vaccination. In 2010, the Saudi Food and Drug Authority approved bivalent and quadrivalent vaccines (Cervarix and Gardasil, respectively) to prevent HPV in Saudi Arabia, and in 2019 the Ministry of Health allocated vaccinations for females aged 11–26. Yet, there is still a lack of vaccination coverage. In addition, there are insufficient studies evaluating the efficacy and safety of vaccines (4), and according to a study by Taebi et al. (5), there is a poor knowledge about the HPV vaccine among women, medical students, and nurses.

In Saudi Arabia, social media is becoming a primary sources of HPV awareness among the younger population. Adults in Saudi Arabia are not currently fully aware of STDs and HPV (6). There are limited national and international studies conducted to analyse the population's understanding of HPV infection and their perceptions and attitudes toward HPV vaccination. Studies conducted in Saudi Arabia such as in Riyadh, Jazan, Hail and the western region, reveal that educated individuals have insufficient knowledge about HPV and the importance of vaccination (6–9).

### 1.2 Research problem

The understanding of female nursing students' knowledge and attitudes regarding HPV and vaccination is of great importance. These students belong to a vulnerable age group and need to be educated about HPV and HPV vaccines. As females, they are also an essential part of the community and should be well-informed about HPV and HPV vaccines. Moreover, as future nurses, they play a crucial role in stressing the burden of disease associated with HPV and advocating for vaccination programs. Raising awareness about HPV is expected to encourage vaccination uptake, which could contribute to a reduction in infection rates. This study is particularly significant within the framework of the Kingdom of Saudi Arabia (KSA)'s Vision 2030, which prioritizes enhanced human health as a pivotal objective.

Recent work at Umm Al-Qura University (UQU) has begun to map students' understanding of human papillomavirus (HPV) and its vaccine. Gari et al. (10) surveyed 479 female medical students and reported that while a large majority recognised cervical cancer (86%), only 40.7% had even heard of HPV and just 30.5% were aware of the vaccine. Actual uptake was negligible (2.1%), despite 72% expressing willingness to be vaccinated. Another study was conducted Sindi (11) surveyed 596 female under-graduates from several Makkah universities—most of them at UQU—and found that 58% achieved an adequate knowledge score, with 61% willing to accept vaccination. Fear of side-effects and needle anxiety were the main barriers. The study recommended comprehensive awareness drives that tackle misconceptions and build confidence in the national immunization program.

Taken together, these two studies show that, even in a health and medical colleges, many students remain poorly informed about HPV and only modestly inclined to act on the information they do possess. Both studies highlight the mismatch between positive attitudes and real-world vaccination rates, underscoring the need for educational strategies that turn theoretical acceptance into preventive action.

The current study extends this evidence base in three important ways. First, it focuses on nursing students—the future front-line educators who will counsel patients and administer vaccines—rather than on medical students or a mixed female cohort. Second, by relating knowledge scores to demographic variables (e.g., living arrangements, parental education) the study pinpoints specific sub-groups that require tailored interventions. These contributions not only deepen understanding of HPV awareness within the university but also translate findings into concrete curriculum recommendations aimed at closing the knowledge–action gap identified by previous researchers.

### 1.3 Research aim

This study aims to assess the level of knowledge and attitudes toward human papillomavirus (HPV) infection and vaccination among female nursing students at Umm Al-Qura University in Makkah, Saudi Arabia.

### 1.4 Research questions

1. What is the level of knowledge about HPV among female nursing students at Umm Al-Qura University?
2. What are the attitudes of female nursing students at Umm Al-Qura University towards HPV vaccination?
3. Are there significant differences in the understanding of HPV among female nursing students at Umm Al-Qura University based on demographic variables such as age, academic year, and prior healthcare experience?

## 2 Literature review

Human papillomavirus (HPV) constitutes a significant global public health concern, encompassing more than 100 viral strains, several of which are oncogenic and responsible for cervical, genital, and oropharyngeal cancers (1). Transmission primarily occurs through sexual contact. Despite widespread prevalence, public knowledge and awareness of HPV infection and vaccination remain limited—particularly in societies where sexual health discussions are considered culturally sensitive (12).

A Saudi study by Alqarawi et al. (13) reported alarmingly low awareness levels, with only 13.7% of 387 surveyed women demonstrating basic knowledge about HPV. Similarly, Al-Qasem et al. (14) found that among 1,065 women attending a gynecology clinic in the Aseer region, only 21.6% were aware of HPV vaccination, and 15.4% recognized its link to cervical cancer. These findings reflect a substantial knowledge gap in the general population.

Jradi and Bawazir (9) further explored cultural barriers in screening and vaccination among Saudi women and healthcare workers. Their study revealed that 30% of healthcare workers lacked preventive knowledge, and 63.3% had never undergone screening, often due to the absence of clear institutional rationale. This underscores the importance of culturally sensitive educational campaigns to enhance screening and vaccination adherence.

In addition, several studies highlight the minimal awareness of HPV among university students. Altamimi (8) found that only 15.3% of Saudi female students were aware of cervical cancer, and merely 7.9% recognized HPV as a causative factor. Alshammari and Khan (6) reported that only 18.4% of students in Hail were familiar with HPV transmission routes, while 61.9% were aware of vaccines and 27.2% understood that HPV can be asymptomatic. These results collectively emphasize the need for structured educational initiatives across academic institutions.

Among nursing students specifically, Abdelaliem et al. (15) found that 73.5% had limited or no knowledge of HPV, and only 26.5% demonstrated adequate understanding. Similarly, Shakurnia et al. (16) observed significant disparities between nursing and medical students' awareness levels, attributing these gaps to sociocultural taboos surrounding immunization and sexual health topics.

Beyond the student population, parental knowledge also influences HPV vaccine uptake. Alkalash et al. (2) reported that only 7.2% of parents had vaccinated their children, with poor understanding of infection risk as the main barrier. Conversely, parents with higher knowledge levels were more likely to exhibit positive attitudes toward vaccination.

Several recurring barriers have been documented in the literature, including misconceptions about vaccine safety, doubts about efficacy, and cost concerns (17, 18). These misconceptions contribute to negative perceptions and hinder vaccine acceptance. Despite moderate to positive attitudes observed in some groups, persistent cultural and informational barriers limit vaccine uptake.

In summary, the reviewed literature consistently highlights a limited understanding of HPV infection and vaccination across different regions of Saudi Arabia. While studies from Riyadh, Jazan, and Hail have contributed valuable insights into general awareness among women and university students, the focus has largely remained outside the Makkah region. Moreover, most previous investigations have concentrated on medical or general female student populations, leaving nursing students who serve as future educators and frontline advocates for preventive health underrepresented in the existing evidence.

This lack of data represents a clear research gap, particularly given the crucial role of nursing students in shaping community health behaviors and supporting vaccination initiatives. The present study seeks to address this gap by examining the knowledge and attitudes of female nursing students at Umm Al-Qura University in Makkah toward HPV infection and its vaccination. Findings from this research are expected to provide region-specific evidence that can guide curriculum development and public health strategies aimed at improving HPV awareness and vaccine acceptance in western Saudi Arabia.

## 3 Methodology

### 3.1 Research approach/design

The study design for this study was descriptive cross-sectional study using a quantitative method was conducted in the female nursing faculty of Umm Al-Qura University in Makkah, Saudi Arabia.

### 3.2 Population and sampling

The target population was a total of 561 female undergraduate nursing students enrolled at Umm Al-Qura University. Among this a total of 261 participants were recruited using a non-probability purposive sampling technique. The sample size was calculated through the Raosoft calculator (http://www.raosoft.com/samplesize.html), assuming a 50% expected knowledge prevalence, 95% confidence level, and 5% margin of error. The required minimum sample size was 229 students, which was exceeded to enhance representativeness.

### 3.3 Inclusion and exclusion criteria

The inclusion criterion was being a nursing student willing to participate in the study. This applied to nursing students from the first to the fourth year of study, and nursing students enrolled in internships at Umm Al-Qura University. Male nursing students, master's students and students from other faculties were excluded from participating in the study.

### 3.4 Data collection

Data were collected over a 3-week period from October 8 to October 26, 2023 using an online questionnaire created with Google Forms. The survey link was distributed electronically to female nursing students via the Nursing Student Affairs Office at Umm Al-Qura University. The recruitment email included information about the study purpose, eligibility criteria, and voluntary participation, along with a secure link to the questionnaire. Two reminder emails were sent during the data-collection period to encourage participation.

Out of a total population of 561 eligible female nursing students, 261 valid responses were obtained, resulting in a response rate of 46.5%. The questionnaire, titled “Knowledge and Attitudes Toward HPV and Its Vaccination for University Students”, contained 28 items and required approximately 10–15 min to complete. It was originally developed by Stridh and Hammar (19) and later adapted to the Saudi cultural context by removing or rewording items that were not suitable or relevant.

The instrument comprised three main parts: The first part gathered demographic information, including participants' age, academic level, and parents' educational background. The second part assessed general knowledge about HPV, divided into two sections. Section A contained four closed-ended questions answered using a nominal scale, while Section B included nine statements rated as true, false, or I don't know. Correct answers received one point, while incorrect or “I don't know” responses received zero. Scores were categorized as poor (0–2), moderate (3–6), or high (7–9). The third part measured attitudes toward HPV vaccination, consisting of yes/no questions on awareness and vaccine history, and additional items rated on a four-point Likert scale (strongly agree, agree, disagree, strongly disagree).

To minimize response bias, all participants were assured that their responses would remain anonymous and confidential, and no personal identifiers were collected. The online form permitted only one submission per participant. In addition, participation was completely voluntary, and respondents were informed of their right to withdraw at any time before submitting the form. These measures helped promote honesty and reduce social-desirability bias.

### 3.5 Validity and reliability

The original version of the questionnaire was validated by two subject-matter experts, an oncology nurse and a cervical cancer specialist, who reviewed the tool for clarity, relevance, and cultural appropriateness (19). Based on their feedback, several items were revised or removed, and the content validity index (CVI) for the knowledge section was 0.80, indicating a good level of expert agreement.

In this study, the instrument was further reviewed for cultural suitability, and its internal consistency was reassessed using data from the current sample. Although the original validation reported a Cronbach's alpha of 0.75, a pilot test with 10% of the current participants (*n* = 26) was conducted to re-evaluate reliability after adaptation. The recalculated Cronbach's alpha (0.702) indicates acceptable internal consistency for social-science research, confirming the tool's suitability for use among Saudi nursing students.

### 3.6 Data analysis methods

In this study, data were analyzed using SPSS version 28. The analysis included several components, such as demographic data and measures to determine the knowledge and attitudes of nursing students toward HPV. Descriptive statistics, including frequencies, percentages, means, and standard deviations, were used to summarize the data. Internal consistency of the questionnaire was evaluated using Cronbach's alpha (*α*).

Before conducting inferential analyses, the assumptions of normality and homogeneity of variance were examined. Normality was assessed through visual inspection of histograms and Q–Q plots, which showed that the data were approximately normally distributed. One-way ANOVA tests were performed to assess differences in knowledge and attitudes among participants. Alongside *p*-values, effect sizes (eta squared, *η*^2^) were calculated to enhance the interpretability of the findings. The effect sizes indicated small to moderate effects (*η*^2^ = 0.016–0.042) across the analyzed variables. Statistical significance was set at *p* < 0.05.

### 3.7 Ethical considerations

The ethics certificate for the research and IRB approval were obtained from the Biomedical Research Ethics Committee of Umm Al-Qura University on September 19, 2023 (approval number HAPO-02-K-012-2023-09-1742), along with official permission to conduct the study (Supplementary Appendices C,D). Permission to use the questionnaire was also granted by the original tool's adaptor.

Electronic informed consent was obtained from all participants before data collection. The consent form was embedded at the beginning of the online questionnaire, clearly outlining the study purpose, voluntary participation, confidentiality assurances, and the right to withdraw at any time without consequences. Participants were required to click “I agree to participate” before accessing the survey, and no personal identifiers such as names, student numbers, or email addresses were collected.

All responses were automatically recorded anonymously and stored in a password-protected Google Drive folder accessible only to the primary researcher. Data were later transferred to a secured, encrypted file on the researcher's personal computer to prevent unauthorized access. The researchers strictly adhered to ethical principles of autonomy, beneficence, and justice to ensure participants' rights, safety, and fair treatment throughout the study.

## 4 Research findings

### 4.1 Demographic data

As shown in Table 1, the demographic characteristics of the participants (*N* = 261) revealed that the mean age was 21.1 ± 1.8 years, with most students (92.7%) aged between 16 and 23 years. Regarding academic year, 22.6% were in the first year, 33.3% in the second, 6.1% in the third, 35.2% in the fourth, and 2.7% were interns. The majority (89%) lived with their families, and most participants (90.0%) had not received the HPV vaccine. About half of the participants' parents held a college or university degree.

**Table 1:** Demographic information of the study participants (*N* = 261).

Items	Categories	*f*	%
Age			
	16–23	242	92.7
	24–31	6	2.3
	32–39	13	5
Which year of college are you currently in?			
	1st year	59	22.6
	2nd year	87	33.3
	3rd year	16	6.1
	4th year	92	35.2
	5th year (Internship)	7	2.7
With whom do you live?			
	Parents/family	232	89
	Friend	4	1.5
	Relatives	5	1.9
	By myself	16	6.1
	Others	4	1.5
Mother's Education			
	Primary School	39	14.9
	Secondary School	28	10.7
	High School	50	19.2
	College or University	135	51.7
	None	9	3.4
Father's Education			
	Primary School	19	7.3
	Secondary School	37	14.2
	High School	75	28.7
	College or University	118	45.2
	None	12	4.6
Have you received the HPV vaccine?			
	Yes	26	10.0
	No	235	90.0

### 4.2 Knowledge and awareness of HPV

As shown in Table 2, most participants (86.6%) recognized that HPV can cause genital warts and certain types of cancer. However, only 62.8% had heard of HPV previously, and the Internet was identified as the primary information source, followed by radio, friends, and healthcare professionals.

**Table 2:** Level of knowledge about human papillomavirus (HPV) among female nursing students (*n* = 261) (section A).

Items	Categories	*f*	%
The human papillomavirus (HPV) can cause genital warts and some cancers			
	Yes	226	86.6
	No	35	13.4
Before today, have you heard of HPV?			
	Yes	164	62.8
	No	97	37.2
From where did you get information about HPV? mark one or two			
	Friends	29	12.7
	Television	23	10.0
	Radio	34	14.8
	Internet	41	17.9
	Brochure	28	12.2
	Magazine	20	8.7
	Public education	18	7.9
	Health professionals (doctor, nurse, midwife, etc.)	22	9.6
	Not sure	238	91.2

According to Table 3, approximately half of the respondents correctly identified HPV as a sexually transmitted infection and as a cause of cervical cancer. More than half (52.5%) were uncertain whether HPV could be cured by antibiotics, and only 11.9% correctly knew that HPV can heal spontaneously. Awareness of available screening methods was limited, with 57.5% acknowledging that a simple vaginal test can detect HPV.

**Table 3:** Level of knowledge of human papillomavirus (HPV) among female nursing students (*n* = 261) (section B).

Statements (the correct answer)	True (1 mark)	False (0 mark)	I don't know (0 mark)
	*f* (%)	*f* (%)	*f* (%)
HPV can cause cervical cancer (true)	130 (49.8%)	10 (3.8%)	121 (46.3%)
HPV is a sexually transmitted disease (true)	139 (53.2%)	33 (12.7%)	89 (34%)
HPV can be cured by taking antibiotics (false)	55 (21%)	69 (26.4%)	137 (52.5%)
HPV can heal by itself (true)	31 (11.9%)	114 (43.7%)	116 (44.4%)
Cervical cancer is the most common cancer among women in Saudi Arabia (false)	68 (26%)	75 (28.7%)	118 (45.2%)
There is a simple vaginal test to find out if you have HPV (true)	150 (57.5%)	13 (5%)	98 (37.5%)
It is important for women to be screened for HPV (true)	195 (74.7%)	10 (3.8%)	56 (21.4%)
Most people with genital HPV infection have visible signs or symptoms (false)	95 (36.4%)	42 (16%)	124 (47.5%)
HPV is very common (true)	78 (29.9%)	54 (20.7%)	129 (49.4%)

Table 4 presents the distribution of total knowledge scores, which ranged from 0 to 9. Nearly half of the participants (49.4%) had poor knowledge, 47.1% had moderate knowledge, and 3.4% demonstrated high knowledge levels.

**Table 4:** Knowledge scores of the participants (*N* = 261).

Poor (score: 0–2)	*f* (%)	Moderate (score: 3–6)	*f* (%)	High (score: 7–9)	*f* (%)
*0	104 (39.84%)	3	29 (11.11%)	7	5 (1.9%)
1	6 (2.29%)	4	36 (13.79%)	8	4 (1.5%)
2	19 (7.27%)	5	36 (13.79%)	9	0 (0)
		6	22 (8.42%)		
Total	129 (49.43%)		123 (47.13%)		9 (3.44%)

### 4.3 Attitudes toward the HPV vaccine

As shown in Table 5, participants generally demonstrated positive attitudes toward HPV vaccination. Most agreed or strongly agreed that they would take the vaccine if it were free (46.75% and 40.23%, respectively). Similarly, most participants (57.08%) expressed a strong desire for more information about HPV and vaccination.

**Table 5:** Attitudes of Umm Al-Qura University female nursing students towards the HPV vaccine (*N* = 261) (section A).

Item	Strongly agree	Agree	Disagree	Strongly disagree
I think my parents/guardians could pay for the vaccine.	94 (36%)	110 (42.15%)	48 (18.39%)	9 (3.45%)
I would get the vaccine if it were free.	122 (46.75%)	105 (40.23%)	23 (8.81%)	2 (0.77%)
I would pay for the vaccine if I could.	86 (32.95%)	115 (44.05%)	55 (21.07%)	5 (1.92%)
I would be embarrassed to ask my parents/guardians about vaccination.	49 (18.77%)	67 (25.67%)	92 (35.25%)	53 (20.31%)
It is not necessary for me to get the vaccination.	41 (15.71%)	73 (27.97%)	110 (42.15%)	37 (14.18%)
If I get the vaccination, it is not necessary for me to be protected against other sexually transmitted infections.	35 (13.41%)	76 (29.12%)	75 (28.74%)	75 (28.74%)
My parents would not allow me to get the vaccine.	34 (13.03%)	49 (18.77%)	113 (43.30%)	65 (24.90%)
I wish to get more information about HPV and HPV vaccination.	149 (57.08%)	84 (32.18%)	26 (9.96%)	2 (0.77%)

However, some students reported social or familial hesitation. Around 25.67% agreed and 18.77% strongly agreed that they would feel embarrassed to ask their parents about vaccination. The mean attitude score, shown in Table 6, was 2.79 ± 0.87, indicating a moderate to slightly positive overall attitude toward HPV vaccination.

**Table 6:** Frequency and percentage distribution of responses for each item related to the attitudes towards the HPV vaccine (*N* = 261) (section A).

Item	Strongly agree	Agree	Disagree	Strongly disagree
I think my parents/guardians could pay for the vaccine.	94 (36%)	110 (42.15%)	48 (18.39%)	9 (3.45%)
I would get the vaccine if it were free.	122 (46.75%)	105 (40.23%)	23 (8.81%)	2 (0.77%)
I would pay for the vaccine if I could.	86 (32.95%)	115 (44.05%)	55 (21.07%)	5 (1.92%)
I would be embarrassed to ask my parents/guardians about vaccination.	49 (18.77%)	67 (25.67%)	92 (35.25%)	53 (20.31%)
It is not necessary for me to get the vaccination.	41 (15.71%)	73 (27.97%)	110 (42.15%)	37 (14.18%)
If I get the vaccination, it is not necessary for me to be protected against other sexually transmitted infections.	35 (13.41%)	76 (29.12%)	75 (28.74%)	75 (28.74%)
My parents would not allow me to get the vaccine.	34 (13.03%)	49 (18.77%)	113 (43.30%)	65 (24.90%)
I wish to get more information about HPV and HPV vaccination.	149 (57.08%)	84 (32.18%)	26 (9.96%)	2 (0.77%)

### 4.4 Differences in knowledge and attitudes by demographic characteristics

As shown in Table 6, the ANOVA results indicated no significant differences in HPV understanding based on year of study (*F* = 1.126, *p* = 0.343, *η*^2^ = 0.016), mother's education (*F* = 1.411, *p* = 0.231, *η*^2^ = 0.020), or father's education (*F* = 1.210, *p* = 0.306, *η*^2^ = 0.016).

However, Table 7 shows that there was a significant difference in HPV understanding based on living situation (*F* = 2.826, *p* = 0.025, *η*^2^ = 0.042), where students living with parents or family members achieved higher knowledge scores than those living independently.

**Table 7:** Mean and standard deviation for various statements related to the students’ attitudes toward the vaccine (*N* = 261) (section B).

Statements	Mean	SD
I think my parents/guardians could pay for the vaccine.	3.11	.820
I would get the vaccine if it were free.	3.33	.717
I would pay for the vaccine if I could.	3.08	.783
I would be embarrassed to ask my parents/guardians about vaccination.	2.43	1.015
It is not necessary for me to get the vaccination.	2.45	.921
If I get the vaccination, itis not necessary for me to be protected against other sexual transmitted infections.	2.27	1.018
My parents would not allow me to get the vaccine.	2.20	.960
I wish to get more information about HPV and HPV vaccination.	3.46	.698
Total	2.79	0.87

The effect sizes (*η*^2^ = 0.016–0.042) for ANOVA tests indicated small to moderate magnitudes, suggesting that while statistically significant, group differences were limited in practical terms.

## 5 Discussion

The results show that a significant portion of the participants (62.8%) had heard of HPV before the day of the study, indicating some level of prior awareness. It is worth noting that our study findings contradict the results of a study conducted by Abdelaliem et al. (15) in Saudi Arabia, where 73.5% of nursing students knew little to nothing about HPV. This discrepancy may be attributed to various factors; for example, the cultural context of the two studies may have influenced the level of awareness, as attitudes towards sexual health and discussions about HPV may vary across different regions and communities.

Our study sheds light on the sources of information for nursing students regarding HPV. The findings indicate that the Internet and social media were the most common sources of information. This aligns with the increasing role of digital platforms in disseminating health-related information, particularly among younger populations. The accessibility and convenience of online sources may explain their popularity among nursing students. However, it is important to consider the quality and accuracy of information obtained from the Internet and social media, as misinformation and misconceptions about HPV can easily spread through these channels. In contrast to our findings, a study conducted by Altamimi (8) in Hail found that healthcare professionals were considered the most reliable sources of information for nursing students. This difference in findings may be attributed to the availability and accessibility of healthcare professionals. In addition, the emphasis on their role in health education may differ between our setting and the setting in the study by Altamimi (8).

The findings of this study indicate that a significant proportion of nursing students (49.4%) had a poor level of knowledge about HPV. This result is contradictory to previous studies conducted in Spain and Indonesia, which reported a moderate level of knowledge about HPV and HPV vaccination among nursing students (20, 21). This aligns with findings from previous studies that reported comparable gaps in HPV knowledge among university students (32). However, it aligns with the findings of Altamimi (8) and of studies conducted in South Carolina and Albania, which identified knowledge gaps among university students (22, 23). Additionally, studies carried out in China, India and Algeria (24–26) revealed significant knowledge gaps regarding HPV and HPV vaccines among women, which supports our findings.

The discrepancies between the findings of this study and those of previous research conducted in Spain and Indonesia may be due to differences in educational curriculum and teaching methods, cultural and societal factors, and the context in which the studies were conducted. The consistent findings between this study and those conducted in South Carolina and Albania suggest that knowledge gaps among university students are not limited to a specific geographical location, thus highlighting the need for improved education and awareness regarding HPV and HPV vaccines among university students globally.

There were notable knowledge gaps in other areas. For instance, a significant proportion of participants were unaware that HPV cannot be cured by taking antibiotics or that it can heal by itself. In addition, some were unaware of the availability of a simple vaginal test. Most participants (86.6%) were aware of the association between HPV and genital warts and cancer, and 49.8% specifically answered in the affirmative to the question of whether HPV causes cervical cancer. This is consistent with the results of the study by Shivani and Rajalekshmi (25), in which participants showed a solid understanding of cervical cancer risk factors, signs, symptoms and preventive measures. This is in contrast to a study conducted in Hail that found only 15.4% of participants were aware of the connection between HPV and cervical cancer (14).

The findings emphasize the importance of targeted educational interventions to improve awareness and understanding of HPV, its transmission, prevention, and associated health risks. By addressing these knowledge gaps, healthcare professionals can play a vital role in promoting HPV prevention and early detection strategies, ultimately reducing the burden of HPV-related diseases. This is in line with the study by Bantun et al. (27), which emphasizes increased education and awareness initiatives aimed specifically at Saudi women. Women must be educated about HPV transmission, the risk of cervical cancer, and the cruciality of vaccination.

Regarding attitudes toward HPV vaccination, 55.6% of the students had never heard of the vaccine before the study, and 90.0% had not been vaccinated against HPV. This is consistent with the results of the study by Xie et al. (26), which found that only 47.54% of the women had an acceptable level of knowledge about HPV vaccines. This is also consistent with the study by Shivani and Rajalekshmi (25), the results of which indicated a significant gap in knowledge and awareness about HPV vaccination among the participants, as only 55.9% knew about the vaccine. It is also consistent with the study by Al-Qasem et al. (14) in Saudi Arabia, where only 21.6% of the women surveyed knew about HPV vaccination.

In our study, the students displayed positive attitudes overall. This is a favorable finding, as positive attitudes can encourage vaccine uptake. The students viewed HPV vaccination as a valuable preventive measure. This result aligns with previous research conducted in Italy and South India, which reported high acceptability and intention to receive the vaccine among nursing and medical students (28, 29). It is also consistent with the findings of Abdelaliem et al. (15), which suggested that a majority of nursing students held a moderate attitude towards HPV vaccination. Similarly, Darraj et al. (4) found that a significant percentage of respondents expressed a desire to receive the HPV vaccine and intended to recommend it to others. However, some studies, such as that by Turki and Alqurashi (17), have reported negative attitudes towards the HPV vaccine, with a considerable proportion of participants holding inaccurate perceptions about cervical cancer.

Despite the positive attitudes observed, there are concerns and potential barriers associated with HPV vaccination. These include issues related to obtaining parental consent and communication challenges. Students had slightly negative attitudes about discussing vaccination with their parents or guardians, which may be attributed to parental consent being required for minors to receive certain vaccines. This requirement poses a significant barrier, particularly if parents are hesitant about vaccination. In the Saudi cultural context, such hesitation may stem from taboos surrounding discussions of sexual health and a lack of open dialogue between parents and children about reproductive health. Cultural modesty and fear of social judgment may prevent families from acknowledging HPV as a relevant health issue. These barriers highlight the need for culturally sensitive public-health communication strategies that respect local values while providing clear, science-based information. Campaigns should focus on family-centered education, using trusted figures—such as community leaders, healthcare professionals, and educators—to normalize conversations about HPV and vaccination. Additionally, integrating HPV education into school-based health programs and premarital counseling could help bridge the communication gap and reduce parental resistance. Communication challenges may involve difficulties in discussing the topic with parents or healthcare providers. This finding is consistent with a study conducted in Poland, which found that low parental knowledge and attitudes acted as significant barriers to vaccination (30). Similarly, a study conducted in Saudi Arabia revealed that only 7.2% of parents vaccinated their children against HPV (2), further supporting our study findings.

These findings highlight the importance of addressing these concerns and providing comprehensive education about the benefits and importance of the HPV vaccine to improve acceptance and uptake among female nursing students. To increase HPV vaccination rates, it is recommended that these concerns and barriers be addressed through targeted interventions and educational campaigns. Providing more information about HPV and the vaccine is essential and will enable students and their parents to make informed decisions. It is important to note that there was variability in attitudes among participants, suggesting that not all students had the same level of positivity or the same concerns regarding HPV vaccination. Some students have strong positive attitudes, while others have more reservations. Tailoring interventions and communication strategies to address the specific needs of different groups is crucial to bridge the knowledge gap.

The study also explored the potential influence of demographic characteristics, such as college year, living situation and parental education levels on students’ understanding of HPV. The results demonstrated that there were no substantial disparities in knowledge and understanding across different college years. This non-significant result may reflect a uniform level of HPV awareness across all academic years, possibly due to the similarity of the nursing curriculum, which does not progressively expand HPV-related content in higher levels. Another explanation could be that overall awareness remains generally low among students, regardless of their academic progression. Similar patterns have been reported in previous Saudi and international studies, where minimal curricular variation or insufficient emphasis on sexual health topics limited students' differential knowledge gains (15, 23). This finding suggests a need to integrate HPV and reproductive health education more explicitly and progressively throughout the nursing curriculum.

This finding contradicts the study by Sallam et al. (31), which suggested that older participants and medical students were more likely to receive the HPV vaccine. This suggests that the educational curriculum at the university helps in effectively maintaining a consistent baseline of knowledge about HPV among nursing students. However, it also raises the question of whether the educational programs are providing enough opportunities for knowledge growth.

The lack of significant differences in HPV knowledge across college years may not necessarily be a positive outcome. Ideally, one would expect students to acquire more in-depth knowledge and expertise regarding HPV as they advance in their nursing education. If this is not happening, it may be necessary to review the curriculum and ensure that students are adequately prepared for their future roles in healthcare. It is important to note that while this result suggests no significant differences, it does not provide insight into the absolute level of HPV knowledge among students. It is possible that the overall understanding of HPV is either very high or very low but consistently so across different college years. Further research is needed to determine the actual level of knowledge among students and whether it aligns with the desired level of understanding in the healthcare field.

Additionally, the statistical analysis showed significant differences in the understanding of HPV among students based on their living situations. Students living with their parents or family exhibited notably higher levels of knowledge compared with those living independently or with peers. Factors related to students' living situations—such as family dynamics, access to information, and cultural influences—may have influenced their understanding of HPV. These findings align with regional and international studies emphasizing the role of social and family environments in shaping health awareness. In Saudi Arabia, Alkalash et al. (2) and Al-Qasem et al. (14) found that students who lived with family members demonstrated higher levels of health literacy and vaccine awareness, likely due to parental guidance and open discussions about preventive health. Similarly, research in Poland (30) and India (25) reported that individuals living in close family settings were more likely to access accurate health information and maintain positive attitudes toward HPV prevention compared with those living alone. In contrast, a study conducted in China (26) found that students living away from their families had lower vaccination awareness, suggesting that social support and familial communication play a vital role in shaping preventive health behaviors. These comparisons highlight the importance of considering family engagement and living context when designing educational interventions to promote HPV awareness and vaccination among university students.

This suggests the need for educational efforts tailored to specific student groups to enhance their understanding of HPV. The finding of distinct differences based on living arrangements underscores the importance of tailoring educational efforts to specific student groups. For example, if students living with their parents or family tend to have a higher level of HPV understanding, it might be valuable to use this insight to design educational interventions or materials that could also benefit students living independently or in other situations. These tailored efforts could help bridge the knowledge gap and ensure that all students receive the necessary information about HPV. Understanding how living arrangements influence HPV knowledge is not only relevant for educational purposes but also for public health initiatives. It could inform strategies to disseminate information and promote HPV vaccination more effectively among groups that might have limited knowledge due to their living situations.

Furthermore, the study found no statistically significant differences in students' understanding of HPV based on their mothers' or fathers' education levels. This lack of association between parental education and students’ knowledge may indicate that HPV awareness is shaped more by institutional and societal exposure than by family background. In Saudi culture, discussions related to sexual and reproductive health are often limited regardless of parents' education level, which could explain the uniformity in knowledge. This implies that formal nursing education and public-health communication, rather than family influence, serve as the main channels for HPV information among young adults.

This suggests that parental education background may not significantly impact students' knowledge of HPV. These findings contradict the study by Smolarczyk et al. (30), which reported that parental education levels influenced children's attitudes towards HPV vaccination. This result indicates that students from various backgrounds, regardless of their parents' education levels, have a similar level of understanding when it comes to HPV. This finding highlights the importance of equal educational experiences for all students, irrespective of their family backgrounds, thus promoting educational equity.

### 5.1 Integration of theoretical context

The observed discrepancy between the students' generally positive attitudes and the low rate of actual HPV vaccination can be interpreted through the Health Belief Model (HBM). According to this model, individuals' health behaviors are influenced by their perceptions of susceptibility, severity, benefits, and barriers. In this study, although nursing students expressed favorable attitudes and recognized the benefits of vaccination, perceived barriers—such as cultural sensitivity, parental consent, and limited accessibility—likely outweighed perceived benefits. Furthermore, low perceived susceptibility to HPV infection, due to cultural taboos and misconceptions about risk, may have reduced motivation to act.

This theoretical framing suggests that educational and public-health interventions should not only provide factual knowledge but also address psychological and sociocultural barriers. Communication strategies that enhance perceived susceptibility, clarify benefits, and reduce perceived barriers may help translate positive attitudes into preventive behavior and higher vaccine uptake.

## 6 Limitations

This study has several limitations that should be considered when interpreting the results.

First, the questionnaire used was originally developed by Stridh and Hammar (19) and was later adapted and culturally modified for the Saudi context. Although every effort was made to ensure clarity and cultural appropriateness, some sensitive terms related to sexual health may still have influenced participants' understanding of certain items.

Second, the study was conducted at a single site—Umm Al-Qura University—and included only female nursing students, which limits the generalizability of the findings to male students and to nursing populations in other institutions or regions of Saudi Arabia.

Third, the data were collected through self-reported responses, which may have introduced recall or social desirability bias, as participants could have provided answers they perceived as acceptable rather than fully accurate.

Finally, the cross-sectional design captures knowledge and attitudes at one point in time and therefore cannot determine cause-and-effect relationships between variables.

Future research should include multiple universities, both male and female nursing students, and longitudinal designs to provide a more comprehensive understanding of how cultural, social, and educational factors influence HPV knowledge and vaccine attitudes over time.

## 7 Conclusion

This study showed that nursing students at Umm Al-Qura University have only a basic level of knowledge about Human Papillomavirus (HPV), even though they generally hold positive attitudes toward vaccination. The gap between their willingness and their actual knowledge highlights a challenge that could limit their ability to counsel patients and promote vaccination effectively in future clinical practice. Living arrangements were found to influence knowledge, while socioeconomic background had little impact, suggesting that access to accurate information plays a more important role than personal background in shaping awareness.

Based on the study findings, a three-tier strategy is recommended to address these gaps and strengthen HPV-related education among nursing students:

- Curricular Integration: Include HPV-related topics across all nursing courses and add simulation-based workshops to enhance students' counselling and communication skills.
- Structural Support: Organize on-campus vaccination drives and focused seminars for students with lower knowledge levels to help turn positive attitudes into real vaccination uptake.
- Future Research: Conduct studies across multiple universities and over longer periods to track knowledge retention and explore social and cultural factors that influence students' willingness and decisions to receive the HPV vaccine.

Ultimately, these steps can help prepare future nurses to serve as effective educators and vaccine advocates within the Kingdom's cervical cancer prevention efforts.

## Data Availability

The original contributions presented in the study are included in the article/Supplementary Material, further inquiries can be directed to the corresponding author.
