# Supplementary material for: Assessment of knowledge and attitudes toward human papillomavirus and its vaccination among female nursing students at Umm Al-Qura University, Saudi Arabia
Source: Front Glob Womens Health. 2025 Dec 18;6:1669950. doi: 10.3389/fgwh.2025.1669950 (PMC12756078; doi:10.3389/fgwh.2025.1669950)
Supplement: Supplementary file 1 [file Datasheet1.pdf]

Kingdom of Saudi Arabia
Ministry of Education
Umm Al-Qura University
031

المملكة العربية السعودية
وزارة التعليم
جامعة أم القرى
٠٣١

1st October 2023

To:
Vice Dean for Academic Affairs
College of Nursing
Umm Al-Qura University

Subject: Approval to Distribute Research Questionnaire to Female Nursing Students

Dear Colleague,

As the Dean of the College of Nursing at Umm Al-Qura University, I am pleased to inform you that approval has been granted to Ms. **Mona Alwadai**, master's student at the College of Nursing, to conduct her research entitled:

"Evaluation of Female Nursing Students' Knowledge and Attitude about Human Papillomavirus (HPV) and Vaccination at Umm Al-Qura University in Makkah, Saudi Arabia."

This study has been reviewed by Biomedical Research Ethics Committee at Umm Al-Qura University and received the Approval No. (**HAPO-02-K-012-2023-09-1742**) on **19/09/2023.**

I kindly request your full cooperation in facilitating the distribution and completion of the questionnaire during an appropriate time that does not interfere with scheduled academic activities.

Thank you for your continued support and collaboration.

Sincerely,

Associate Prof. Fatimah Jaber Alsolami

Dean, College of Nursing
Umm Al-Qura University
Makkah, Saudi Arabia

Ministry Of Education
Umm Al-Qura University
وزارة التعليم
جامعة أم القرى
كلية التمريض
Faculty of Nursing
