# Supplementary material for: Assessment of knowledge and attitudes toward human papillomavirus and its vaccination among female nursing students at Umm Al-Qura University, Saudi Arabia
Source: Front Glob Womens Health. 2025 Dec 18;6:1669950. doi: 10.3389/fgwh.2025.1669950 (PMC12756078; doi:10.3389/fgwh.2025.1669950)
Supplement: Supplementary file 2 [file Datasheet2.pdf]

Kingdom of Saudi Arabia
Ministry of Education
Umm Al-Qura University
031

المملكة العربية السعودية
وزارة التعليم
جامعة أم القرى
٠٣١

Approval No. (HAPO-02-K-012-2023-09-1742)

FINAL APPROVAL FROM THE BIOMEDICAL RESEARCH ETHICS COMMITTEE

Principal Investigator: **Nawal Gamel Abdulghani**	Faculty**: Nursing**	Date: **19/09/2023**

Proposal Title (in English):

Evaluation of Female Nursing Students Knowledge and Attitude about Human Papillomavirus (HPV) and Vaccination at Umm Al-Qura University in Makkah, Saudi Arabia.

The Biomedical Research Ethics Committee has evaluated and examined the above-mentioned research proposal and has found it to be in accordance with the specifications and conditions of the ethics of scientific research.

The Committee has accordingly granted the Principal Investigator final approval concerning the ethics of scientific research

Principle Investigator is permitted to:

- Initiate the implementation of scientific research procedures within faculty facilities and laboratories, in addition to the regional research centers and hospitals
- Publish in scientific journals

Responsibility of Principal Investigator:

- Must provide a written statement to the Vice presidency of post-graduate studies and scientific research regarding any changes in the research plan, the committee shall decide whether a new approval is needed or not.

Director of Biomedical Ethics Committee

Dr. Aiman M. Momenah
Umm Al-Qura University

لجنة الأخلاقيات الحيوية - جامعة أم القرى - وزارة التعليم
Biomedical Ethics
Ministry Of Education-Umm Al-Qura University

المرفقات:
التاريخ: 19/09/2023
الرقم: DOOS170923
